# Roles of MicroRNA-34a in Epithelial to Mesenchymal Transition, Competing Endogenous RNA Sponging and Its Therapeutic Potential

**DOI:** 10.3390/ijms20040861

**Published:** 2019-02-16

**Authors:** Dongsong Nie, Jiewen Fu, Hanchun Chen, Jingliang Cheng, Junjiang Fu

**Affiliations:** 1 Department of Chemistry and Chemical Engineering, Hunan Institute of Science and Technology, Yueyang 414006, China; 12005030@hnist.edu.cn; 2 Key Laboratory of Epigenetics and Oncology, the Research Center for Preclinical Medicine, Southwest Medical University, Luzhou 646000, China; fujiewen@swmu.edu.cn; 3 Department of Biochemistry and Molecular Biology, School of Life Sciences, Central South University, Changsha 410013, China; chenhanchun@csu.edu.cn

**Keywords:** microRNA-34a, epithelial-mesenchymal transition, competing endogenous RNA, circular RNA, p53

## Abstract

MicroRNA-34a (miR-34a), a tumor suppressor, has been reported to be dysregulated in various human cancers. MiR-34a is involves in certain epithelial-mesenchymal transition (EMT)-associated signal pathways to repress tumorigenesis, cancer progression, and metastasis. Due to the particularity of miR-34 family in tumor-associated EMT, the significance of miR-34a is being increasingly recognized. Competing endogenous RNA (ceRNA) is a novel concept involving mRNA, circular RNA, pseudogene transcript, and long noncoding RNA regulating each other’s expressions using microRNA response elements to compete for the binding of microRNAs. Studies showed that miR-34a is efficient for cancer therapy. Here, we provide an overview of the function of miR-34a in tumor-associated EMT. ceRNA hypothesis plays an important role in miR-34a regulation in EMT, cancer progression, and metastasis. Its potential roles and challenges as a microRNA therapeutic candidate are discussed. As the negative effect on cancer progression, miR-34a should play crucial roles in clinical diagnosis and cancer therapy.

## 1. Introduction

MicroRNAs (miRNAs or miRs) are a class of high-conserved, single-stranded noncoding RNAs that are small, only 20–24 nucleotides in length. They can bind to 3′-untranslated regions (UTRs) of messenger RNAs (mRNAs) to either inhibit mRNA translation or induce mRNA degradation or deadenylation, thus silencing gene expression at the posttranscriptional level [1,2]. Since Lee et al. [3] discovered the first miRNA lin-4 in 1993, many miRNAs have been revealed, and the functions of miRNAs have gradually been clarified. MiRNAs have been reported to control about 30% of fundamental gene expressions in humans, most of which are essential for normal survival and development [4,5,6]. Therefore, by regulating the target gene expression, miRNAs can be involved in various kinds of signaling pathways to modulate many important biological processes, including cellular proliferation, metastasis, apoptosis, senescence, differentiation, autophagy, and immune responses [7]. miRNAs have been found to be dysregulated under pathological conditions, such as neurodegenerative diseases, cardiovascular diseases, autoimmune diseases, and cancers [8,9,10]. The functions of miRNAs may serve as tumor suppressors to inhibit tumor cell proliferation or as oncogenes (oncomiRs) to induce tumorigenic processes [8,11,12]. 

MicroRNA 34a (miR-34a), as a member miR-34 family (miR-34a/b/c, miR-34s) [13,14], has been reported to be dysregulated in various cancers, and is the first miRNA that was demonstrated to be directly regulated by p53 [15]. Although the expression of miR-34a includes endogenous expression or mimics transfection, the mature miR-34 has been shown to be inactive in several cancer cells given the lack of a 5′-phosphate. When DNA-damaging stimulus is applied to these cells, inactive miR-34a can be rapidly activated through 5′-end phosphorylation [16]. The dysregulation of miR-34a in cancers has been the focus of researchers. This dysregulation of miR-34a has been reported in many different cancers, including colorectal cancer, prostate cancer, breast cancer, lung cancer, liver cancer, and osteosarcoma, as well as other diseases. Numerous studies showed that miR-34a could be a potential biomarker for diagnosis and prognosis in different types of cancers [17,18,19,20,21,22]. A recent systematic review and meta-analysis verified the diagnostic value of miR-34a in detecting breast cancer [23]. Primary tumor cells can acquire migratory and invasive abilities through epithelial to mesenchymal transition (EMT) and form metastases [24,25,26,27]. A large quantity of experimental data showed that miR-34a can influence EMT in metastatic cancers. Competing endogenous RNA (ceRNA) is a new hypothesis that states that mRNA, circular RNA (circRNA), pseudogene transcript, and long noncoding RNA (lncRNA) regulate each other’s expressions using microRNA response elements (MREs) to compete for the binding of miRNAs [28,29].

In this review, we focus on the functions of miR-34a in cancer and its underlying EMT mechanisms. ceRNA is introduced to discuss the novel miR-34a regulation in EMT and cancer progression. MiR-34a’s promising therapeutic potential and the challenges faced are also discussed.

## 2. Underlying EMT Mechanism of MiR-34a in Cancer Metastasis

**Figure 1:**
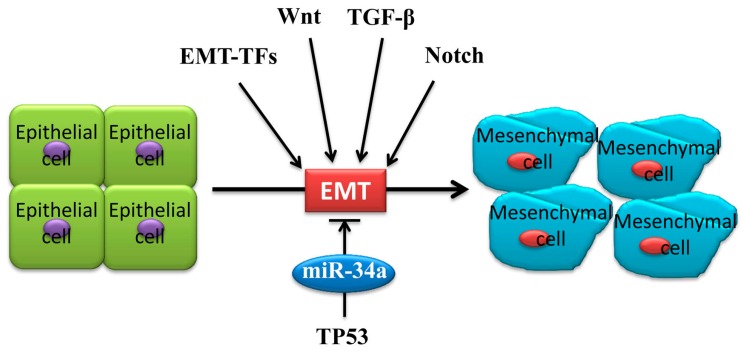
Mechanisms of epithelial to mesenchymal transition (EMT) regulation. EMT is characterized by loss of cell polarity and a decreased expression of some epithelial markers, such as E-cadherin, cytokeratin, and α-catenin, as well as an increased expression of some mesenchymal markers, such as N-cadherin, vimentin, fibronectin, and matrix metalloproteinases (MMPs). As a cellular biological process, epithelial cells lose their morphologies and adhesion abilities and gain a mesenchymal phenotype and increased motilities. Signaling pathways for EMT-TFs, Notch, Wnt, and TGF-β, were regulated by miR-34a for tumor-associated EMT. Note: EMT-TFs, Epithelial to mesenchymal transition inducing transcription factors; TGF, transforming growth factor.

Most tumor cells achieve metastatic and invasive ability through EMT, resulting in a poor prognosis and even death [30]. As a cellular biological process, epithelial cells lose their morphologies and adhesive abilities and gain a mesenchymal phenotype, such as increased motilities (Figure 1) [31,32]. Thus, EMT is characterized by a loss of cell polarity and a decrease in expression of some epithelial markers, such as E-cadherin, cytokeratins, and α-catenin, as well as an increase in expression of some mesenchymal markers, such as N-cadherin, vimentin, fibronectin, and matrix metalloproteinases (MMPs) [24,30,31,33]. An increasing number of findings has documented the negative effect of miR-34a in tumor cell proliferation, metastasis, and invasiveness, indicating the important relationship between miR-34a and tumor-associated EMT [34,35].

## 3. MiR-34a Regulates EMT in Cancer Cells

**Figure 2:**
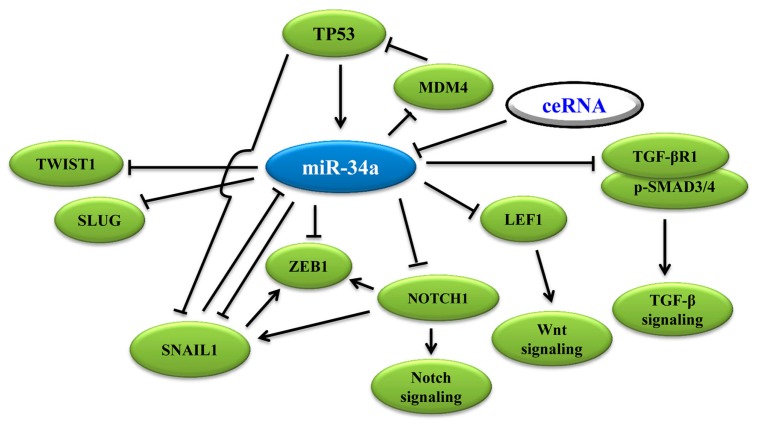
MiR-34a-controlled signaling for EMT. MiR-34a inhibits EMT-TFs, Notch signaling, Wnt signaling, and TGF-β signaling, whereas TP53, miR-34a, and NOTCH1 form feedback loops with each other for EMT. The competing endogenous RNAs (ceRNAs) is a novel concept about mRNAs, circRNAs, pseudogene transcripts and lncRNAs that regulate each other’s expression to compete for the binding of miRNA. Note: arrow indicates promotion, whereas t-bar indicates inhibition.

EMT can be categorized using physiological tissue contexts. The most well-defined type is EMT in tumor growth and cancer progression (type 3 EMT) [36], when cancer cells at the invasive front of the tumors convert into a mesenchymal phenotype [33]. EMT generates cells with invasive properties that enable them to move into the blood stream and spread systemically to other organs. EMT is an important process in tumor evolution, providing the possibility for tumor cells to adapt to the tumor’s microenvironment. Appropriately cellular environments, cytokines, and extracellular signals may induce EMT [37,38,39]. One study showed that inducing the expression of pri-miR-34a using doxycycline could result in the down-regulation of vimentin and the upregulation of E-cadherin in human colon cancer cell SW480 [40], suggesting that miR-34a can negatively regulate EMT to inhibit proliferation and invasion. In addition to the expression change in EMT markers, the deregulating expression of EMT-associated transcription factors (EMT-TFs), such as SLUG, SNAIL, and ZEB1, were also observed [41,42]. EMT-TFs are also essential to the activation of tumor-associated EMT [43] (Figure 1). A systematic review and meta-analysis suggested that the overexpression of TWIST1, SNAIL1, and especially SLUG plays a key role in the aggregation of metastatic breast cancer (MBC) treatment as well as in the improvement of follow-up plans in MBC patients [44]. A wealth of data indicated that microRNAs can bind with EMT-TFs, thus forming double-negative feedback loops to interfere with tumor-associated EMT [42,45]. Besides EMT-TFs, miR-34a can also control EMT via other approaches. In short, miR-34a plays its tumor-suppressive role as a vital negative regulator for EMT in tumors. Figure 2 summarizes miR-34a-controlled signaling in tumor-associated EMT in cancer cell progression.

### 3.1. MiR-34a Binds to 3′-UTR of EMT-TFs to Regulate EMT

Three positive EMT-TFs are most promising: (1) zinc-finger transcription factors SNAIL family, including SNAIL1, SNAIL2, and SNAIL3 (also known as SNAIL, SLUG, and SMUC, respectively); (2) ZEB transcription factors, including ZEB1 and ZEB2; and (3) basic helix-loop-helix (bHLH) transcription factors family, including TWIST1, TWIST2, and E12/E47 [41,46,47]. EMT-TFs are necessary for the activation of EMT (Figure 1). MiR-34a binds to 3′-UTR of EMT-TFs directly to regulate tumor-associated EMT. 

SNAIL 3′-UTR was reported to contain a conserved sequence that matches miR-34 [36,40,48,49]. The SNAIL family is the only EMT-TF that has a matched sequence with all three miR-34 family members (miR-34a/b/c). The dual-reporter assay further demonstrated that SNAIL is the direct target of the miR-34 family [48,49]. In addition to the SNAIL family, UTRs of other EMT-TFs, such as ZEB1, ZEB2, and TWIST1, also exist in conserved sequence(s) that match miR-34a, as well as the stemness factors BMI1, CD44, CD133, and c-MYC [13,36,42,50], which are down-regulated by direct miR-34a binding. Thus, miR-34a can also down-regulate the stemness factors BMI1, CD44, CD133, OLFM4, and c-MYC [40,51]. These above studies clearly showed the inhibition of miR-34a on EMT-TFs, which results in the attenuation of EMT.

Among these EMT-TFs, SNAIL and TWIST1 are especially unique because they can combine with the E-box sequences of E-cadherin promoter to suppress the expression of E-cadherin, leading to the strengthening of EMT [32,52,53]. SNAIL can enhance the expression of mesenchymal genes, like vimentin in ovarian cancer [54] and matrix degradation enzyme MMP9 in hepatocellular carcinoma (HCC) [55]. Apart from regulating the expression of related epithelial and mesenchymal genes, SNAIL also has a positive effect on other EMT-TFs, TWIST1, and ZEB1 [55]. 

Loss-of-function or mutation in p53 promotes cancer cell EMT by de-repressing SNAIL1 protein expression and activity [48]. Activation of p53 has been shown to down-regulate SNAIL via induction of the miR-34a/b/c. Suppression of miR-34a/b/c causes up-regulation of SNAIL; cells displayed EMT marker changes and related features, therefore enhancing migration and invasion. Ectopic miR-34a expression induces mesenchymal-epithelial-transition (MET) and down-regulates SNAIL expression [40]. SNAIL and ZEB1 bind to E-boxes in the miR-34a/b/c promoters, thereby repressing miR-34a/b/c expression. Thus, by shifting the equilibrium of these reciprocal regulations toward a metastatic state, p53, SNAIL1, and miR-34 form a feedback loop to control the initiation of cancer cell EMT program [40,48]. Imani et al. reported that the co-delivery of miR-34a and thymoquinone (TQ), a small molecular component from *Nigella sativa*, inactivates the downstream of the EMT signaling by targeting EMT-TFs, TWIST1, and ZEB1 [13,42].

### 3.2. MiR-34a Induces p53 Activation via Targeting TP53 and MDM4

In addition to p53 regulating the expression of miR-34a, miR-34a has been shown to induce p53 activation by directly targeting TP53 and MDM4, a strong p53 transactivation inhibitor [14,56]. MiR-34a contributes to p53 function by targeting multiple p53 inhibitors, including the histone deacetylases SIRT1 and HDAC1, and MTA2 that deacetylates p53, thereby epigenetically increasing the transcriptional activity of p53 [57,58,59,60]. The complexity in both the positive and negative effects of miR-34a on the p53 network suggests that rather than simply promoting the p53 response, miR-34a might stabilize the robustness of the p53 response to genotoxic stress [56].

Compared with p53 wild type cells, miR-34s were down-regulated in p53-mutated ovarian cancer cells [61]. When cells were treated with Nutlin-3a, a chemical activator of p53, the expression of miR-34a significantly increased [62]. This research indicated that miR-34a expression follows the change in p53, and demonstrated that miR-34a is a downstream target of p53. More importantly, p53 has been reported to diminish the EMT progress by moderating SNAIL expression and activity by strengthening the expression of miR-34s [48]. Collectively, miR-34a, p53, and EMT shape an intricate network to influence each other’s functioning (Figure 2) [35].

### 3.3. MiR-34a Regulates EMT via Wnt, TGF-β1/Smad3/4, Notch Signaling and Others

MiR-34a regulation of tumor-associated EMT can modulate not only by EMT-TFs and tumor suppressor p53, but also some fundamental signal pathways, such as Wnt [63,64], TGF-β1/Smad3 (transforming growth factor-beta 1/Smad3), and Notch1 [65,66] (Figure 2). 

### 3.4. Wnt Signaling Pathway 

A study indicated that miR-34a could negatively control Wnt transcriptional activity by regulating multiple pathway-associated genes in both mammary glands and breast cancer [67]. Expression of miR-34a significantly decreased in patients with hepatitis B virus (HBV)-activated liver fibrosis and hepatocellular carcinoma (HCC), as well as in CC14 induced liver fibrosis model mice. 

Lymphoid enhancer-binding factor-1 (LEF1) is an important TF in the Wnt signaling pathway, involved in regulation of cell proliferation and invasion. MiR-34a modulates the levels of LEF1 to regulate EMT in prostate cancer cells. Functionally, miR-34a is negatively correlated with the migration and invasion of prostate cancer cells through LEF1. Likewise, miR-34a could be involved in the Wnt pathway by specifically repressing LEF1 to regulate the EMT process in prostate cancer [68]. miR-34a contributes to the chemosensitivity of BIU87/ADR, an epirubicin (EPI) chemoresistant cell line, by inhibiting the TCF1/LEF1 axis in bladder cancer [69].

The HOX transcript antisense RNA (HOTAIR), a lncRNA, is highly abundant and conserved, and it has been implicated in many essential biological processes and diseases, including cisplatin (DDP) resistance in gastric cancers. HOTAIR knockdown inhibited DDP resistance by upregulating miR-34a, indicating that the effect of HOTAIR/miR-34a axis on gastric cancer (GC) cells is involved in the PI3K/Akt and Wnt/β-catenin signaling pathways [70].

### 3.5. Notch Signaling Pathway 

In addition to EMT-TFs, miR-34a can modulate EMT by binding to pivotal target genes, such as NOTCH1 in Notch signaling [71]. For example, miR-34a can bind to the 3′-UTR of Notch1 and Jagged1 in colon cancer cells and cervical cancer cells, thus inhibiting the cell migratory ability and the expression of vimentin and fibronectin, and promoting the expression of E-cadherin [65,66,71]. Treatment options for metastatic castrate-resistant prostate cancer (mCRPC) are limited and typically centered on paclitaxel-based chemotherapy. Liu et al. [72] found that miR-34a attenuates chemoresistance to paclitaxel by regulating target genes, *JAG1* and *Notch1*, which are associated with drug resistance. NFIX circular RNA (circNFIX), which regulates NOTCH1 to promote glioma progression by sponging miR-34a-5p via the Notch signaling pathway, will be discussed in the latter portion of this review [73].

### 3.6. TGF-β1 Signaling Pathway 

Transforming growth factor-beta1 (TGF-β1) is a type of secretive protein affecting the same cells that secrete the protein (autocrine) or its neighboring cells (paracrine). TGF-β1, a key member in the TGF-β superfamily, can be pro-tumorigenic or tumor suppressive. TGF-β induction of EMT is considered a pro-tumorigenic state. Huang et al. [74] revealed that miR-34a is able to reverse TGF-β-induced EMT, invasion, and migration by suppressing Smad4 in nasopharyngeal carcinoma cells (NPC). 

### 3.7. Other Pathways 

As a target of miR-34 family members, interleukin-6 receptor (IL-6R) can mediate the activation of signal transducer and activator of transcription 3 (STAT3), whereas the oncogenic transcription factor STAT3 can bind to miR-34a via a conserved binding site to repress the expression of miR-34a. The inhibition of miR-34a is essential for IL-6-induced EMT and invasion [75]. Thus, IL-6R/STAT3/miR-34a constitutes a feedback loop to regulate EMT for suppressing tumor progression that may be relevant for future therapeutic approaches [76].

## 4. CeRNA in Cancer Progression: A New Pattern of Gene Expression Regulation Sponging MiR-34a

**Figure 3:**
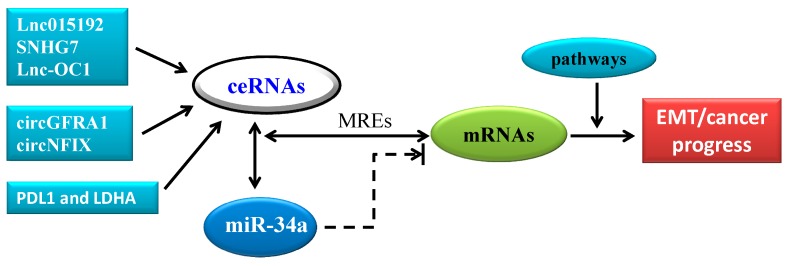
CeRNA in cancer progression: a new pattern of gene expression regulation sponging miR-34a. The lncRNAs, including lnc015192, SNHG7, and lnc-OC1; circ RNAs including circGFRA1 and circNFIX; and coding RNA including PDL1 and LDHA, can function as ceRNAs through MREs to regulate target gene expression by sponging miR-34a, for EMT in cancer progression and metastasis. Note: solid arrow indicates the major pathways, whereas dashed arrow indicates another regulatory pathway.

The competing endogenous RNA (ceRNA) is a novel concept about mRNA, circRNA, pseudogene transcripts, and lncRNAs regulating each other’s expression using microRNA response elements (MREs) to compete for the binding of miRNAs [28,29,77,78] (Figure 2). Although purely new, the conception of ceRNA can be traced to the 2007 study by Ebert et al. [79]. Numerous lines of evidence in bioinformatics, cell biology, and animal models have supported the ceRNA hypothesis for EMT in cancer progression [78,80,81,82] (Figure 3).

LncRNAs, defined as RNAs of more than 200 nucleotides in length, have been implicated in a variety of disease states. Mounting evidence shows that lncRNAs can function as ceRNAs for miRNAs in distinct pathological states. Huang et al. [83] reported that Adam12 and lnc015192 promote breast cancer metastasis partly by sponging miR-34a through the ceRNA mechanism. First, they found that after knocking out miR-34a in breast tissues, Adam12 and lnc015192 were significantly upregulated. Then, knockdown Adam12 and lnc015192 inhibited breast cancer cell migration, invasion, and EMT. Further experiments revealed that lnc015192 regulates Adam12 expression by functioning as a ceRNA for miR-34a. SNHG7 is another lncRNA that can promote tumor growth and EMT via regulating miR-34a [84]. Li et al. [85] revealed that SNHG7 facilitates proliferation and metastasis as a ceRNA to regulate GALNT7 expression through sponging miR-34a in colorectal cancer (CRC) progression by playing an oncogenic role in regulating the PI3K/Akt/mTOR pathway by competing with endogenous miR-34a and increasing GALNT7. Tao et al. [86] indicated that Lnc-OC1 promotes cell proliferation and migration by sponging both miR-34a and another miR-34 family member, miR-34c, in ovarian cancer. In addition to cancer progression, lncRNA also plays a role in other diseases. For example, UFC1 was reported to promote proliferation of chondrocyte in osteoarthritis by acting as a sponge for miR-34a [87].

Circular RNA (circRNA) is an another novel type of RNA recently discovered which, unlike the better known linear RNA, forms a covalently closed continuous loop. In circular RNA, the 3′ and 5′ ends normally presenting in an RNA molecule are joined together [88,89,90]. In addition to protein-coding potential, endogenous circular RNAs are resistant to exonuclease-mediated degradation and are presumably more stable than most linear RNAs in cells given the lack of its 5′ or 3′ ends [91,92]. circRNA enrichment in the cytoplasm, coupled with extensive complementarity of circRNAs to their linear mRNA counterparts, shows the possibility of these RNAs exerting their functional roles through microRNA binding. Thus, circRNAs have potentially important roles in gene regulation [89,93], such as ciRS-7, which could serve as miRNA sponges [94]. circRNAs have been shown to regulate EMT, indicating that some circRNAs may affect EMT-related cellular functions including carcinogenesis and metastasis [95]. Very recent studies showed that circRNAs act as miR-34a sponges in cancers. He et al. [96] indicated that circGFRA1 and GFRA1 act as ceRNAs in triple negative breast cancer (TNBC) by regulating miR-34a. MiR-34a has also been shown to directly suppress lactate dehydrogenase A (LDHA) in colorectal cancer and breast cancer [97] as well as PDL1, an important immune checkpoint inhibitor, in lung cancer and acute myeloid leukemia [98]. Further study revealed that both PDL1 and LDHA acted as ceRNAs that promote proliferation and metastasis of TNBC by regulating miR-34a [99]. Thus, simultaneous targeting PDL1 and LDHA, combined with immunotherapy and metabolically targeted treatments, might represent a new breast cancer treatment, especially in TNBC. By sponging miR-34a-5p via the Notch signaling pathway, another circRNA, circNFIX, was reported to regulate NOTCH1 to promote glioma progression [73]. CircNFIX is overexpressed in glioma, and the Notch signaling pathway is considerably upregulated in tumor tissues compared with paired normal brain tissues by acting as a sponge in miR-34a that targets NOTCH1. Si-circNFIX and miR-34a mimic promote cell apoptosis, whereas a miR-34a inhibitor can neutralize the suppressive effect of si-circNFIX on glioma cells. In vivo experiments demonstrated that si-circNFIX suppresses glioma cell growth by regulating miR-34a and Notch signaling.

Taken together, these studies clearly indicate that ceRNA plays a vital role in cancer progression and/or EMT through gene expression regulation by sponging miR-34a.

## 5. MiR-34a as a Promising Agent for MicroRNA Therapeutics

Insights into the roles of miRNAs in development and disease, particularly in cancer, have highlighted miRNAs as potential tools and targets for novel therapeutic approaches [11,100]. Due to their dysregulation in cancers, miRNAs are classified into two types: tumor suppressors or oncogenes (oncomiRs) [12]. According to these two distinct functions of miRNAs in cancer, an innovative therapeutic approach that relies on miRNAs was introduced [5,101]. This novel therapeutic approach uses miRNA mimics or antimiRs to modulate miRNA expression and activity in vivo [11,102,103]. 

Metformin, a drug approved by the U.S. Food and Drug Administration as a prescription medication to treat diabetes, was revealed to have an anticancer function. Wang et al. [104] investigated metformin’s regulatory role of the (SNAIL/miR-34):(ZEB/miR-200) system for CRC therapy in the EMT process. They assessed its anti-EMT abilities and explored its inherent pharmacological mechanisms for CRC therapy. Metformin inhibited proliferation, migration, and invasion in CRC cells by up-regulation of E-cadherin and down-regulation of vimentin, thereby exhibiting anti-EMT characteristics. In this system, miR-200a, miR-200c, and miR-429 levels increased and miR-34a, SNAIL1, and ZEB1 levels decreased in the TGF-β-induced EMT after metformin treatment. Divergent with the main topic of this review, metformin decreased miR-34a but exhibited anti-EMT effects; thus, metformin may bidirectionally regulate the (SNAIL/miR-34):(ZEB/miR-200) system for CRC therapy. A combination treatment of miR-34a mimic and TQ might enhance their therapeutic effects in targeting EMT-TFs signaling [13,42].

To some extent, miRNA therapeutics is a form of precision medicine; it is specific to certain sites to control gene expression. However, the problem with this form of medicine is the lack of efficiency in the miRNAs delivery system. RNA is easily degraded by RNase, and RNase is abundant in serum and endocytic compartment in tissues or cells. Therefore, when delivering miRNAs mimics or antimiRs to target cells, ensuring their therapeutic efficiency is challenging. Until now, there were two solutions to overcome this problem: chemically modifying nucleotides to increase stability or applying nanocarrier delivery vehicles to avoid degradation [105,106].

Lin et al. [107] selected the VP16-GAL4-WPRE integrated systemic amplifier (VISA) to construct a non-viral delivery vector. The VISA nanoparticle vector contains a human telomerase reverse transcriptase promoter and was used to encapsulate miR-34a (TV-miR-34a). Breast cancer stem cells (BCSC) that were transfected with TV-miR-34a showed extremely high expression of miR-34a. The oncogenic properties and tumor proliferation characteristics of BCSC were inhibited by TV-miR-34a. In a model of HCC, delivery of miR-34a in a polymer-based nanosystem vector resulted in decreased cell proliferation and tumor growth [108]. These two experiments demonstrated the efficiency of miR-34a in oncotherapy. miR-34a therapeutics also produced incredible success in a classical Non-Small Cell Lung Cancer (NSCLC) mouse model that is resistant to conventional anticancer therapy. Using this mouse model, one study, which combined miR-34a with let-7b in a neutral lipid emulsion or a NOV340 nanoparticle, successfully induced miR-34a expression and increased survival [109]. A combination treatment with miR-34a and let-7b could strengthen the anti-proliferative effects of erlotinib in NSCLC cells [110]. Amphiphilic nanocarrier-induced modulation of PLK1 and miR-34a can improve therapeutic responses in pancreatic cancer [111]. In this study, a combination of microRNA and siRNA was delivered by an efficient nanocarrier to pancreatic ductal adenocarcinoma (PDAC) tumors. Using proteomic-microRNA profiles and survival data of PDAC patients from TCGA, a novel signature for prolonged survival had been discovered. Accordingly, a microRNA-mimic to increase miR-34a, together with siRNA to silence PLK1, was combined in in vivo dual-targeting experiments, resulting in the development of a biodegradable amphiphilic polyglutamate amine polymeric nanocarrier (APA). Polyplexes of APA-miRNA-siRNA were systematically administered to orthotopically inoculate PDAC-bearing mice, and they showed no toxicity to normal cells but accumulated inside the tumors, thus showing enhanced antitumor effects. 7C1, a nanoparticle agent, has been used to efficiently deliver miR-34a systematically in mouse models, indicating that the tumor progression was attenuated. The anticancer effect became more prominent with a combination treatment of miR-34a and siRNA-Kras [112]. Depending on nanovector-encapsulated miR-34a mimics, anticancer therapeutics have also been applied in prostate cancer [51,113], neuroblastoma [114], and pancreatic cancer [115]. 

Numerous preclinical studies have shown the broad application of miR-34a in cancer therapeutics, but more articles have been than those already mentioned here. In April 2013, MRX34, a special lipid nanoparticle filled with miR-34 mimics, was tested in clinical trial as the first microRNA-associated therapeutic drug (NCT01829971) in the USA (https://clinicaltrials.gov/ct2/show/NCT01829971) [116]. This trial recruited 155 participants with 7 cancer types including primary liver cancer, SCLC, lymphoma, melanoma, multiple myeloma, renal cell carcinoma, and NSCLC. In August 2016, MRX34 was tested in a clinical trial (NCT02862145) again with advanced melanoma patients (https://clinicaltrials.gov/ct2/show/NCT02862145). Although the trial was quickly stopped because of serious adverse effects, miR-34a therapeutics is worthy of consideration. With increasingly clear understanding concerning miRNAs and miR-34a function, a miR-34a mimic is the most promising microRNA therapeutics.

## 6. Future Outlooks and Challenges

The poor prognosis of cancer is largely ascribed to the metastasis in cancer cells. MiR-34a acts as a negative regulatory factor of tumor-associated EMT and plays a considerable role in repressing tumorigenesis and slowing tumor progression. As a promising tumor suppressor, miR-34a is being considered for cancer therapy. Many studies about miR-34a’s therapeutic potential have been completed and they verified its tumor suppressive role in cancer. However, some challenges emerged with the application of miR-34a therapeutics. One is miRNA degradation, preventing miR-34a from penetrating the capillary endothelium into target cells. The immunoreaction of miR-34a therapeutics also deserves our attention. MRX34 was tested in a clinical trial (NCT02862145), but it was withdrawn because five immune-related adverse events occurred. If miR-34 therapeutics is dependent on nano-vectors, the toxicity of nanoparticles is also worth to discuss. Mechanisms inhibiting therapeutic re-expression of miR-34a in the context of EMT, inflammation, or oncogene signaling could be responsible for insufficient therapeutic effects, whereas aberrant expression of miR-34a in normal cells, or massive necrotic cell death observed in miR-34a-treated tumors, may underlay systemic negative effects of therapy. Even so, other unexpected side effects may occur. Successful management of side effects on non-tumor cells is indispensable for successful therapeutic application. ceRNA’s mechanism is now a novel concept involving the mRNA, circRNA, pseudogene transcripts, and lncRNAs regulation of each other’s expression using MREs to compete for the microRNA binding in EMT and cancer progress. Networks between miR-34a and EMT may be vital diagnostic markers and these networks may be provided therapeutic methods for malignant tumors.
